# Hemodynamic Changes Following Endotracheal Intubation With Glidescope^®^ Video-Laryngoscope in Patients With Untreated Hypertension

**DOI:** 10.5812/cardiovascmed.17598

**Published:** 2014-04-01

**Authors:** Majid Dashti, Shahram Amini, Rasoul Azarfarin, Ziae Totonchi, Maryam Hatami

**Affiliations:** 1 Anesthesiology Department, Sadoughi University of Medical Sciences, Yazd, IR Iran; 2 Anesthesiology Department, Mashhad University of Medical Sciences, Mashhad, IR Iran; 3 Rajaie Cardiovascular and Medical and Research Center, Iran University of Medical Sciences, Tehran, IR Iran; 4 Pain Clinic, Tehran University of Medical Sciences, Tehran, IR Iran

**Keywords:** Airway Management, Intubation, Intratracheal, Hypertension, Hemodynamics, Laryngoscopes

## Abstract

**Background:**

Tracheal intubation can be associated with considerable hemodynamic changes, particularly in patients with uncontrolled hypertension. The GlideScope^®^ video-laryngoscope (GVL) is a novel video laryngoscope that does not need direct exposure of the vocal cords, and it can also produce lower hemodynamic changes due to lower degrees of trauma and stimuli to the oropharynx than a Macintosh direct laryngoscope (MDL).

**Objectives:**

The aim of this clinical trial was to compare hemodynamic alterations following tracheal intubation with a GVL and MDL in patients with uncontrolled hypertension.

**Patients and Methods:**

Sixty patients who had uncontrolled hypertension and scheduled for elective surgery requiring tracheal intubation, were randomly assigned to receive intubated with either a GVL (n = 30) or a MDL (n = 30). Intubation time, heart rate, rate pressure product (RPP), and mean arterial blood pressure (MAP), were compared between the two groups at; baseline, following induction of anesthesia, after intubation, and at one minute intervals for 5 minutes.

**Results:**

A total of 59 patients finished the study. Intubation time was longer in the GVL group (9.80 ± 1.27 s) than in the MDL group (8.20 ± 1.17 s) (P < 0.05). MAP, pulse rate, and RPP were lower in the GVL than the MDL group after endotracheal intubation (P < 0.05). MAP, heart rate, and RPP returned to pre-intubation values at 3 and 4 minutes after intubation in the GVL and MDL groups, respectively (P < 0.05).

**Conclusions:**

Hemodynamic fluctuations in patients with uncontrolled hypertension after endotracheal intubation were lower with the GVL than the MDL technique.

## 1. Background

Airway management in especial circumstances has always been of great significance for physicians of all eras (1). Nowadays, an emphasis is placed on education and proper success in airway management (2) by using the correct instruments. Circulatory response following endotracheal intubation can be significant, and this can result in adverse outcomes particularly in untreated hypertensive patients (2-4). Several drugs are currently used to blunt these effects (4-6); however, they may have deleterious effects on patients' hemodynamics. The Glidescope^®^ video-laryngescope (GVL, Glidescope^®^, Saturn Biomedical Systems Inc. Canada) is a relatively novel device used for both oral and nasal endotracheal intubation in patients with normal and difficult airways (7-9). It has been associated with high success rates and minor complications, even in the hands of novices. Unlike a Macintoch direct laryngoscope (MDL), the GVL has a specially planned blade with a 60° curvature, reducing the uphill lifting force and cervical spine movement necessary to expose the glottis, and that might be the reason it is associated with lower hemodynamic changes. It has been reported that the GVL had no significant advantage over the MDL in decreasing circulatory responses to orotracheal intubation in normotensive patients (10-13). However, evidence is scant about the use of GLV in hypertensive patients, particularly in those who remain untreated.

## 2. Objectives

The aim of this study was to compare hemodynamic changes and intubation time with the GVL and the MDL in patients with untreated hypertension.

## 3. Patients and Methods

After approval by the ethics committee of our institution and obtaining informed consent from all of the patients, 60 patients between the ages of 40 and 60 years, with untreated essential hypertension, who were undergoing elective surgery, were recruited for the study. All patients had visited the pre-operation clinic 1-2 days before their surgery. The patients entered the study when their PB was higher than 140/90 mmHg in both the pre-operation visit and in the second anesthesiologist's visit the night before surgery. The exclusion criteria included: blood pressure more than 180 over 110 mmHg, predicted difficult airway, history of drug abuse, dehydration, history of other cardiovascular diseases, history of consumption of any drugs known to affect the cardiovascular system, diabetes mellitus, and end organ damage due to hypertension. Finally the patients were randomly allocated to either the GVL or the MDL groups using a 'permuted block randomization' method. 

The patients were intubated by a single experienced anesthesiology resident. After oxygenation with 100% oxygen for three minutes, patients were administered midazolam 0.05 mg/kg and intravenous remifentanil infusion 0.1 µg/kg/min, until five minutes after intubation. Anesthesia induction was made with propofol 1.5 mg/kg and cisatracurium 0.15 mg/kg was given to facilitate endotracheal intubation. Ventilation was provided with a face mask with 100% oxygen until full neuromuscular relaxation. Anesthesia was maintained with 60% nitrous oxide in oxygen and propofol 4-6 mg/kg/h to maintain a bispectral index between 40 and 60, and remifentanil 0.1-0.5 g/kg/min to maintain blood pressure and heart rates within 20% of baseline levels. We regarded a difference in MAP of 20% to be clinically important. The tracheal intubation time, defined as the time from grasping the endotracheal tube until passing the tube through the vocal cords, was recorded. The mean arterial pressure (MAP), pulse rates, and rate pressure product (RPP) data, were collected at; baseline, after anesthesia induction, and every minute for 5 minutes after intubation.

A sample size of 28 was needed to obtain a power of 80%, a significance level of 5%, and to detect significant difference of mean arterial pressure (MAP) between the two groups. The collected data were analyzed by using SPSS 15 for Windows (SPSS Inc., Chicago, IL, USA). Continuous variables were presented as mean ± SD. We used one sample Kolmogorov-Smirnov test to compare continuous variables adapting to normal distribution, repeated measures ANOVA for changes within the groups, and chi-square test or Fisher’s exact test as needed for categorical variables. P value≤ 0.05 was considered statistically significant.

## 4. Results

**Figure 1.**
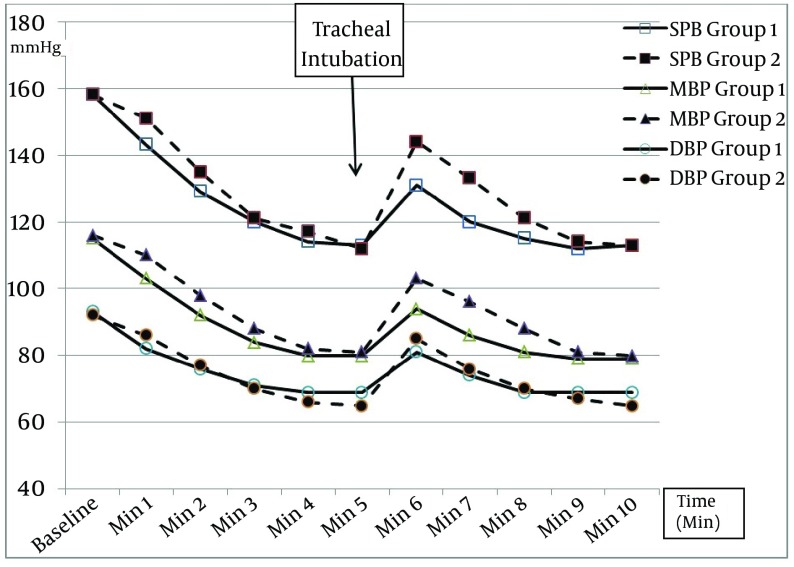
Systolic, Mean and Diastolic arterial Pressure Values During the Study in Both Groups (1 = GVL and 2 = MDL) GVL, GlideScope^®^ video-laryngoscope; MDL, Macintosh direct laryngoscope.

Fifty nine patients completed the study. One patient was excluded in the MDL group because of significant hypotension after induction, requiring drug intervention. The patients were similar in both groups in respect to their demographic data (Table 1). The intubation time was shorter in the MDL group than the GVL group (8.20 ± 1.17 seconds vs. 9.80 ± 1.29; P < 0.01).The mean arterial pressures (MAP) were similar at the baseline in both groups (115.17 ± 6.16 in the MDL and 115.72 ± 7.6 mmHg in the GVL group, P = 0.75). MAP decreased significantly after induction in both groups; however, the percentage of changes were similar in both groups (30.75% in the MDL group and 31.03% in the GVL group; P = 0.72). MAP increase after intubation was significantly higher in the MDL than the GVL group in the first 3 minutes after intubation (Table 2). However, the values returned to pre-intubation levels after 5 minutes in both groups (Figure 1).

**Figure 2.**
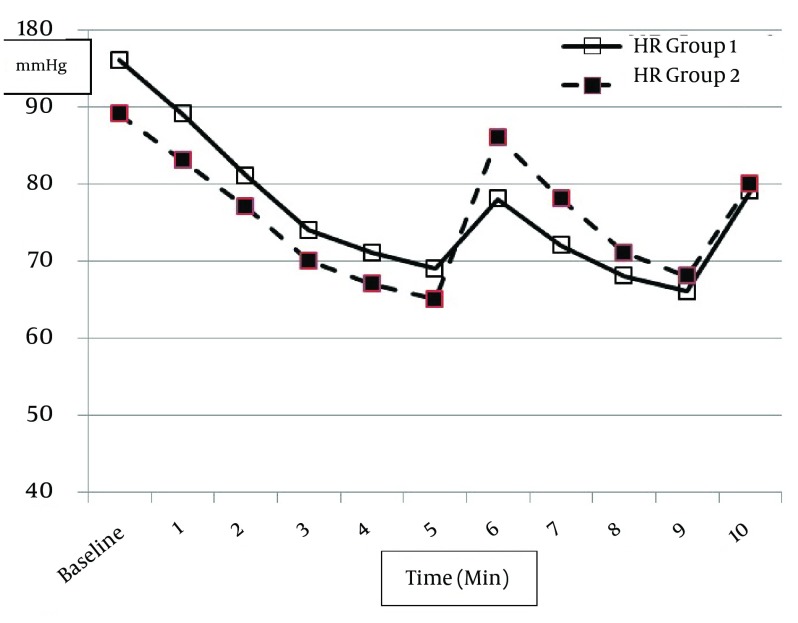
Heart Rate Values During the Study in Both Groups (1 = GVL and 2 = MDL) GlideScope^®^ video-laryngoscope; MDL, Macintosh direct laryngoscope.

The pulse rates and the rate pressure product were significantly higher in the GVL than the MDL group at baseline. PR showed a significant drop after induction in both groups. Nevertheless, this was similar in both groups (Table 3). In contrast, after intubation, the changes were more significant in the MDL than the GVL group in the first 3 minutes (Figure 2). The values returned to pre-intubation levels at 3 and 5 minutes in the GVL and MDL groups, respectively.

**Figure 3.**
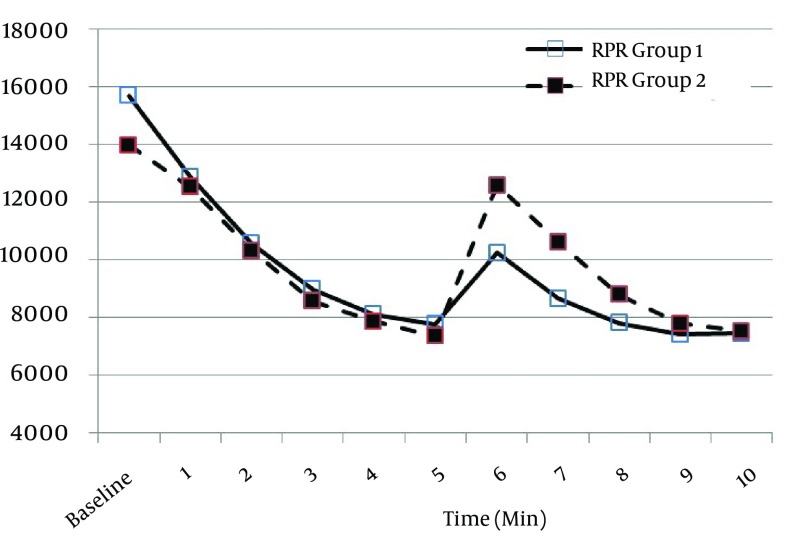
RPR values during the study in both groups (1 = GVL and 2 = MDL) GVL, GlideScope^®^ video-laryngoscope; MDL, Macintosh direct laryngoscope.

RPP decreased markedly after induction, although there were no significant differences between the groups (Table 4). Nevertheless, the increase in RPP after intubation was more significant in the MDL than the GVL group in the first 3 minutes after intubation (Table 4). The RPP returned to pre-intubation levels at 3 and 5 minutes in the GVL and MDL group, respectively (Figure 3).

**Table 1.** Demographic Data of the Patients in the Two Groups ^a,b^

	MDL Group (n=29)	GVL Group (n=30)	P value
**Gender**			>0.05
male	15	19	
female	14	11	
**Age, y**	57.82 ± 4.83	54.82 ± 5.76	>0.05
**Weight, kg**	66.25 ± 6.15	72.14 ± 9.72	>0.05

^a^ GVL, GlideScope^®^ video-laryngoscope; MDL, Macintosh direct laryngoscope; NS, not significant.

^b^ Data are presented as numbers or mean ± SD.

**Table 2.** Mean Arterial Pressures (mmHg) at Baseline, After Induction, and After Intubation in the Study Groups ^a,b^

Timing	MDL Group (n=29)	GVL Group (n=30)	P value
**Basic value**	115.17 ± 6.16	115.72 ± 7.6	0.75
**After induction **	79.43 ± 7.17	80.13 ± 7.85	0.72
**1 min after intubation**	94.30 ± 9.34	105.34 ± 11.14	0.001
**2 min after intubation**	86.23 ± 10.26	96.17 ± 12.04	0.001
**3 min after intubation**	80.73 ± 9.16	87.58 ± 9.68	0.007
**4 min after intubation**	78.73 ± 7.95	81.17 ± 7.07	0.21
**5 min after intubation**	79.10 ± 7.07	80.03 ± 7.07	0.61

^a^ GVL, GlideScope^®^ video-laryngoscope; MDL, Macintosh direct laryngoscope.

^b^ Data are presented as numbers or mean ± SD.

**Table 3.** Heart Rates (beat/min) at Baseline, After Induction, and After Intubation in the Study Groups ^a,b^

Timing	MDL Group (n=29)	GVL Group (n=30)	P value
**Basic value**	99.51 ± 17.17	88.72 ± 16.92	0.01
**After induction**	68.93 ± 5.63	65.48 ± 9.33	0.09
**1 min after intubation**	78.20 ± 7.41	86.37 ± 14.09	0.007
**2 min after intubation**	72.43 ± 6.96	78.41 ± 14.24	0.01
**3 min after intubation**	68.06 ± 6.74	71.48 ± 11.50	0.03
**4 min after intubation**	66 ± 6.42	67.58 ± 10.06	0.47
**5 min after intubation**	66.10 ± 6.54	66.17 ± 9.44	0.97

^a^ GVL, GlideScope^®^ video-laryngoscope; MDL, Macintosh direct laryngoscope.

^b^ Data are presented as numbers or mean ± SD.

**Table 4.** Rate Pressure Product (RPR) at Baseline, After Induction, and After Intubation in Both Groups ^a,b^

Timing	MDL Group (n=29)	GVL Group (n=30)	P value
**Basic Value**	15 678.79 ± 2589.8	13 979.24 ± 2586.9	0.01
**After induction**	7 764.70 ± 798.83	7 350.34 ± 1337.62	0.15
**1 min after intubation**	10 226.90 ± 1213.88	12 562.69 ± 2962.28	0.0001
**2 min after intubation**	8 663.03 ± 1086.96	10 603.41 ± 2839.46	0.001
**3 min after intubation**	7 783.76 ± 882.44	8 773.55 ± 2099.44	0.001
**4 min after intubation**	7 406.46 ± 743.02	7 778.20 ± 1576.51	0.24
**5 min after intubation**	7 446.34 ± 673.90	7 521.65 ± 1354.92	0.79

^a^ GVL, GlideScope^®^ video-laryngoscope; MDL, Macintosh direct laryngoscope.

^b^ Data are presented as numbers or mean ± SD.

## 5. Discussion

Our study revealed that hemodynamic changes during tracheal intubation are less significant with the GVL than the MDL in untreated hypertensive patients. Hypertension increases the perioperative cardiac risk with an odds ratio of 1.31 (14). However, there was little evidence of an association between a preoperative BP reading of less than 180/110 mmHg and perioperative cardiac risk.

It is usually recommended that elective surgery should be postponed in cases of severe hypertension (diastolic BP > 115 mmHg, systolic BP > 200 mmHg) until BP is less than 180/110 mm Hg. Moreover, it is not clear what range of blood pressure fluctuation is acceptable in hypertensive patients. However, it has been shown that perioperative hemodynamic fluctuations occur less frequently in treated than in untreated hypertensive patients (3, 15, 16) and hemodynamic fluctuations increase morbidity. It has been suggested that rapid correction of BP or prevention of increases in heart rate may be all that is required (17, 18). Based on the above recommendations, we allow operations to be performed on untreated hypertensive patients with BP readings less than 180/110 mmHg undergoing minor surgeries. On the other hand, none of our patients had hypertension related to end organ damage or other cardiopulmonary comorbidities. All intubations were performed by a single anesthesiology resident experienced in both devices. This would eliminate any possible bias regarding device application.

Significant hypertension and tachycardia are associated with tracheal intubation under light anesthesia. The magnitude of the response is greater with increasing force and duration of laryngoscopy. Although these changes are usually transient returning to control levels within five minutes, they can result in myocardial ischemia in patients with cardiac disease. Increasing the depth of anesthesia can blunt these deleterious effects, however, changes in the concentration of anesthetic agents in blood and at effector sites occur slowly in relation to the onset and offset of airway stimuli and hemodynamic responses (19, 20).

On the other hand, a pharmacologic intervention might depress the cardiovascular system resulting in undesired hypotension and bradycardia. Another approach to reduce the cardiovascular response to tracheal intubation is to modify the technique of tracheal intubation. The hemodynamic responses to endotracheal intubation are mainly due to oropharyngeal stimulation produced by laryngoscopy and stimulus on the larynx and trachea due to tracheal tube insertion.

The upward lifting force required to expose the glottis during a laryngoscopy is much less with a Glidescope in comparison to a Macintosh laryngoscope (4.9-13.7 N versus 35-47.6 N), and that results in less traction applied to the soft tissues. Therefore, it might be associated with less sympathetic stimulation. It has been suggested that a general anesthesia of standard clinical depth can effectively suppress the vasopressor response, but not the tachycardia related to orotracheal intubation using a Glidescope (9). Rate pressure product (RPP) has been proposed as an index of myocardial oxygen consumption, with values more than 22000 indicating myocardial ischemia (21). Our patients showed an increased RPR at baseline because of their underlying untreated hypertension. All of the study patients experienced an increase in RPP after intubation, which was more significant in the MDL group. However, this index did not exceed 22000 in any patient, and all of the participants left the hospital uneventfully. In contrast to a study by Xue et al. (10, 11), our results demonstrated better hemodynamic stability following oral endotracheal intubation using GVL, than when using MDL. On the other hand, the GVL has been shown to be superior to the MDL for airway management in patients with treated hypertension (13). Nasal intubation using GVL has also been associated with lesser tachycardia, with shorter duration, in comparison to MDL (22).

In summary, we conclude that endotracheal intubation is associated with less significant hemodynamic changes with a GVL than a MDL in untreated hypertensive patients. However, these changes are not clinically significant.
